# Targeting Cell Cycle Checkpoint Kinases to Overcome Intrinsic Radioresistance in Brain Tumor Cells

**DOI:** 10.3390/cancers14030701

**Published:** 2022-01-29

**Authors:** Tijana Vlatkovic, Marlon R. Veldwijk, Frank A. Giordano, Carsten Herskind

**Affiliations:** 1 Cellular and Molecular Radiation Oncology Lab, Department of Radiation Oncology, Universitätsmedizin Mannheim, Medical Faculty Mannheim, Heidelberg University, 68167 Mannheim, Germany; tijana.vlatkovic@medma.uni-heidelberg.de (T.V.); marlon.veldwijk@medma.uni-heidelberg.de (M.R.V.); 2 Department of Radiation Oncology, Center for Integrated Oncology (CIO), University Hospital Bonn, University of Bonn, 53127 Bonn, Germany; frank.giordano@ukbonn.de

**Keywords:** brain tumor, radiation therapy, radiosensitivity, cell cycle checkpoints, checkpoint inhibitor, ATM, ATR, CHK1, CHK2, WEE1

## Abstract

**Simple Summary:**

As cell cycle checkpoint mechanisms maintain genomic integrity, the inhibition of enzymes involved in these control mechanisms may increase the sensitivity of the cells to DNA damaging treatments. In this review, we summarize the knowledge in the field of brain tumor treatment with radiation therapy and cell cycle checkpoint inhibition via targeting ATM, ATR, CHK1, CHK2, and WEE1 kinases.

**Abstract:**

Radiation therapy is an important part of the standard of care treatment of brain tumors. However, the efficacy of radiation therapy is limited by the radioresistance of tumor cells, a phenomenon held responsible for the dismal prognosis of the most aggressive brain tumor types. A promising approach to radiosensitization of tumors is the inhibition of cell cycle checkpoint control responsible for cell cycle progression and the maintenance of genomic integrity. Inhibition of the kinases involved in these control mechanisms can abolish cell cycle checkpoints and DNA damage repair and thus increase the sensitivity of tumor cells to radiation and chemotherapy. Here, we discuss preclinical progress in molecular targeting of ATM, ATR, CHK1, CHK2, and WEE1, checkpoint kinases in the treatment of brain tumors, and review current clinical phase I-II trials.

## 1. Introduction

Brain tumors include primary brain tumors and brain metastases. In this review, we focus on primary brain tumors, of which the most common in adults are gliomas arising from glial cells [1]. Glioblastoma (GBM, previously named ‘glioblastoma multiforme’) is the most aggressive and the most frequent malignant type, accounting for almost half of malignant tumors of the central nervous system [2]. Other types of gliomas include anaplastic astrocytomas, oligodendrogliomas, and oligoastrocytomas [1]. In children, tumors of the central nervous system are the most frequent solid tumors, making up 15–20% of all malignancies. The most common type of brain tumor in children is medulloblastoma (MBM), a tumor of embryonal origin diagnosed in 20% of instances of all pediatric brain tumors. The overall survival of children with medulloblastoma ranges from 30% to over 90%, depending on the tumor’s genotype [3].

The current standard of care treatment of GBM consists of surgical resection, radiotherapy (RT), and chemotherapy with the DNA alkylating agent temozolomide (TMZ) [4]. TMZ is a prodrug that is activated at physiological pH and methylates purine nucleotides, generating O6-methylguanine in DNA [5]. The addition of TMZ has increased the median overall survival of GBM patients from 12.1 months to 14.6 months and 5-year survival from 1.9% to 10% [4,6,7]. However, progress in outcome is hampered by resistance to RT, TMZ, and other cancer therapies.

Ionizing radiation induces DNA double-strand breaks (DSBs), single-strand breaks (SSBs), base damage, and abasic sites. A typical daily dose fraction of RT (2 Gy) induces around 3000 DNA lesions per cell, which is 17 times less than the estimated number of lesions produced daily due to physiological oxidative stress [8]. The vast majority of sparsely distributed SSBs, base damage and abasic sites are rapidly repaired by base excision repair (BER). However, in contrast with other forms of oxidative stress, ionizing radiation produces tracks of spatially correlated ionizations, some of which are close enough to cause more serious DNA damage, such as DSBs and complex lesions. Although up to 98% of DSBs can be repaired, residual irreparable or misrepaired DSBs and complex DNA damage will prevent cell division and cause clonogenic cell death. The majority of DSBs are repaired by nonhomologous end joining (NHEJ), which is fast and active in all cell cycle phases but may introduce small deletions or inserts. A smaller fraction is repaired by homologous recombination (HR), which is error-free but slower and requires a sister chromatid as a template and, therefore, is only active in the late S or G2 phase. Single-strand annealing (SSA) and alternative end joining (alt-EJ) are backup repair mechanisms acting on complex lesions that are not repaired by the two major DSB repair mechanisms but are more error prone [9]. The O6-methylguanine alkylation of DNA induced by TMZ is repaired by a specialized enzyme, O(6)-methylguanine-DNA methyltransferase (MGMT), which is normally expressed at a low level. However, transcriptional repression by methylated CpG sites in the *MGMT* promotor is frequently abrogated in GBM, making *MGMT* methylation status an important prognostic factor.

Recent reviews have described mechanisms and strategies for targeting DNA repair in brain tumors [10,11]. Additional causes of resistance include the blood–brain barrier, heterogeneity of tumor cells and the tumor microenvironment, as well as molecular mechanisms of resistance to specific therapies [12]. The present review will target the DNA damage response (DDR) by inhibiting kinases involved in the S and G2/M checkpoints to increase mitotic failure. However, before discussing this strategy, we will briefly review the heterogeneity of GBM tumors and the role of glioma stem cells (GSCs) in therapy resistance.

## 2. GBM Heterogeneity and GSC-Related Therapy Resistance

Uncontrolled division of tumor cells is made possible by at least some of the well-established hallmarks of cancer [13]. A characteristic of GBM is the heterogeneity of tumor cells between tumors and within individual tumors. Thus, three major subtypes of GBM have been identified [14,15,16,17]: (i) the classical or proliferative subtype with *EGFR* amplification, deletion of the *CDKN2A* site (coding for p16^INK4A^ and p14^ARF^), activation of the RB pathway, wild-type *IDH1*, and *TP53* genes; (ii) the mesenchymal subtype with deletion of *NF1*, activation of the AKT pathway, and mutation in *PTEN*; (iii) the proneural subtype with amplification of *PDGFRA* and mutations in *IDH1* and *TP53*. A potential fourth “neural” subtype showed a gene expression pattern similar to normal brain tissue and has been identified as contamination by normal cells in the tumor microenvironment [17]. In addition to the major characteristics, a number of other genetic and epigenetic changes are associated with each subgroup (see comparative overview [14]). Heterogeneity within individual tumors was demonstrated by single-cell RNA sequencing and revealed expression profiles representing a continuum from stem-like to differentiated cells, which could be classified according to the GBM subgroups above [18]. However, in addition to the dominant signatures, the different tumor subtypes contained cells with signatures characteristic of other subtypes, underlining the complexity and plasticity of GBM tumor cells [19].

GSCs are a slowly proliferating, self-renewing cell population residing in perivascular niches [20]. It is likely that they originate from neural stem cells in the subventricular zone (SVZ) [14], although the plasticity of GBM cells means that genomic instability and the microenvironment may also cause changes in differentiated cells to become stem-like cells [21,22]. A number of factors contribute to therapy resistance of GSC, such as slow proliferation and hypoxia in the perivascular niche, both of which result in radioresistance. Furthermore, GSCs are considered to have enhanced capacity for DSB repair, with not only NHEJ [23] but also error-free HR contributing to resistance [24,25]. In addition, a number of survival pathways are frequently upregulated in GBM, including the PI3K/AKT pathway, which facilitates repair and may be antiapoptotic (reviewed in [26]), as well as the Notch, Hedgehog (Hh), and Wnt pathways that are associated with stem-cell properties and therapy resistance (reviewed in [27]). The PI3K/AKT pathway is activated by Receptor Tyrosine Kinases (RTK), e.g., EGFR, frequently amplified and upregulated or mutated to produce a constitutive active variant GFRvIII present in approximately 30% of GBM. Unfortunately, strategies to inhibit EGFR have shown limited success, partly due to the development of resistance to the inhibitors [28]. Thus, recurrent GBM tumors may show changes in amplification of EGFR and other RTKs, and EGFRvIII expression is frequently lower [29,30]. The reduction or loss of EGFR expression without loss of self-renewal properties is most likely made possible by redundancy and cross-talk of RTK signaling pathways such as IGF-1R driving resistance and mitogenic signaling independently of EGFR [31,32]. Changes in gene expression of GSC are frequently determined at the epigenetic level and may be influenced by cues from the microenvironment [33]. In general, the plasticity of GBM cells makes GSC a moving target [29] which will need a combination of inhibitors of multiple specific pathways to overcome therapy resistance.

The complexity of finding such inhibitor combinations suggests that it may be helpful to explore alternative approaches by targeting essential mechanisms for cell division in combination with irradiation. A promising concept to enhance the effect of RT is to impair the ability of cells to repair DNA and maintain genomic integrity by targeting enzymes involved in the DNA damage response (DDR) [34]. This approach is being pursued for various solid tumors [35], but relatively few studies have investigated brain tumors.

## 3. The DNA Damage Response (DDR)

The DDR is a complex system evolved with the role of preserving genetic information through the generations, and as such, it is crucial for the functionality of normal tissue. The components of the DDR system can be divided into three groups: sensors, signal transducers, and effectors. Sensors are protein complexes that recognize the DNA damage and activate the signal transducers, which transfer the information via a complex intracellular network to the effectors. The effector pathways include proteins involved in cell cycle control, DNA repair, and apoptosis [34].

A cell’s response to various internal and external signals revolves around the p53 protein encoded by the *TP53* gene. The p53 pathway determines the fate of the cell via involvement in DNA repair, induction of permanent or transient cell cycle arrest, and induction of apoptosis. The activity of p53 is dependent on the number of activators and repressors. In the absence of stress, p53 is ubiquitinated and degraded. Different types of stress signal result in post-translational modifications of p53, including acetylation, methylation, phosphorylation, sumoylation, and ubiquitination. In response to cell stress such as telomere shortening and radiation, a specialized kinase, ataxia telangiectasia mutated (ATM), phosphorylates p53, leading to its stabilization and transcriptional activation of p21 (CDKN1A)-mediated cell cycle arrest. Other kinases involved in checkpoint control, such as ataxia-telangiectasia and Rad3-related protein (ATR), checkpoint kinase 1 (CHK1), and checkpoint kinase 2 (CHK2), also target p53 [36].

ATM, ATR, and DNA protein kinase (DNA-PK) are phosphoinositide 3-kinase (PI3K)-related kinases playing a crucial role in the DDR, both in DNA damage repair and the control of cell cycle progression. The activity of these kinases is strictly regulated via the formation of protein complexes. In response to DSBs, ATM and DNA-PK form complexes with MRE11-RAD50-NBS1 (MRN), or Ku70/80 proteins, respectively. ATR is activated by ATR interacting protein (ATRIP), TopBP1, ETAA1, and other activators and interacts with replication protein A (RPA)-coated DNA strands [37].

Therapy resistance in cancers is now generally considered to be related to the presence of stem-like cells showing increased resistance to radiotherapy and chemotherapy [21]. Radioresistance is ascribed mainly to reduced production and increased scavenging of reactive oxygen species (ROS), enhanced DNA repair, and slower proliferation with more quiescent cells. In glioma, the proportion of stem-like cells increases after irradiation, demonstrating their relative radioresistance [38]. This was related to enhanced phosphorylation of checkpoint kinases ATM, CHK1, and CHK2, implicating an important role of the DDR. The MET RTK-signaling pathway leads to activation of Aurora kinase A, ATM, and p21 in glioblastoma stem-like cells resulting in increased repair and reduced apoptosis [39].

## 4. Cell Cycle Checkpoints

Cell cycle checkpoints regulate whether a cell will replicate and divide, thus passing genetic information on to daughter cells [40]. These mechanisms ensure the correct replication of genetic material, its proper distribution to the daughter cells, and maintenance of genomic stability through cell division. Progression through the four phases of the cell cycle (G1, S, G2, M) is strictly regulated by cyclin-dependent kinases (CDKs), which are activated by forming complexes with cyclins that are expressed at varying levels during the cell cycle.

The majority of healthy cells are quiescent in transient or permanent G0 phase maintained by retinoblastoma protein (RB) that binds and inhibits E2F-DP complexes, which are transcription factors regulating the expression of genes required during cell cycle progression [41,42]. RB is unphosphorylated and represses E2F in G0. In the presence of pro-mitotic signals, such as binding of growth factors to their receptors, an intracellular signal cascade activates CDK4 and CDK6, which form complexes with cyclin D (CCND1, CCND2, and CCND3). The cyclin D-CDK4 complex monophosphorylates RB in early G1 without releasing it from E2F [43]. At the end of the G1 phase, CDK2 is activated by CDC25 phosphatase and forms a complex with cyclin E. Cyclin E-CDK2 hyperphosphorylates RB releasing E2F to induce the expression of proteins involved in replication, such as DNA polymerases α, δ and ε, cyclin E, CDK2, CHK1, and many others [41,43].

At the end of the S phase, cyclin E is degraded and replaced by cyclin A forming a CDK2/cyclin A complex, CDK2/cyclin A phosphorylates CDC6 and E2F1, leading to the G2 phase, while the transition to M phase is regulated by activation of CDK1 (previously known as CDC2 and encoded by the *CDC2* gene) and formation of the CDK1/cyclin B complex. The activity of CDK1 is regulated via WEE1 kinase, MYT1, and CDC25. During the M phase, the progress of cell division depends on low levels of CDK1, and cyclin B and is regulated via the spindle assembly checkpoint [34,40,44,45,46,47]. After mitosis, the activity of CDK3/cyclin C may send the cell into G0 arrest [48].

In the presence of DNA lesions, stress-induced cell cycle checkpoints mediate transient arrest providing time for the repair mechanisms to repair the DNA lesions, leading to clonogenic cell survival in the case of a successful repair. By contrast, severely damaged cells may arrest permanently in the form of senescent or prematurely differentiated cells or may die by apoptosis or other forms of cell death, all of which contribute to clonogenic inactivation [34,40,44,45,46,47].

Stress-induced cell cycle arrest in G1 is dependent on the accumulation of p53 [32]. During normal cell cycle progression, p53 is unphosphorylated and, as such, is marked for degradation by binding of the E3 ubiquitin ligase, mouse double minute 2 homolog, MDM2. However, if DNA damage is present, ATM and checkpoint kinase 2 (CHK2) protein kinases phosphorylate p53, causing the dissociation of MDM2 and accumulation of p53, which then functions as a transcription factor inducing the synthesis of cyclin-dependent kinase inhibitor p21 (CDKN1A) and other targets. P21 binds CDK2/Cyclin E and CDK2/Cyclin A complexes and inhibits their activities, causing temporary cell cycle arrest, especially at the G1/S transition [34,40,44,45,46,47]. The p53-dependent G1/S checkpoint is very sensitive, responding to a single DSB, but takes up to 4 h for full activation [49].

**Figure 1:**
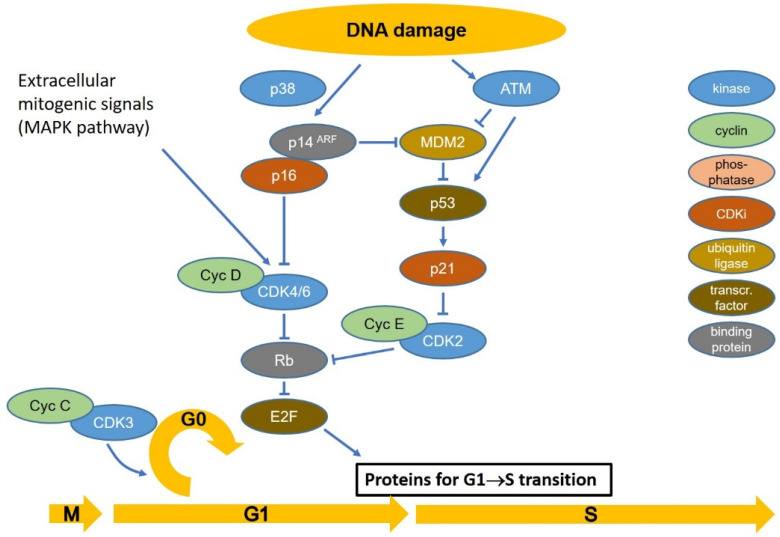
Schematic diagram of the signals regulating the transition of cells from quiescence (G0) to replication in the S-phase and stress-induced arrest at the G1-S transition. Pointed arrows symbolize functional activation, arrows ending with a line symbolize inhibition. In quiescent cells, the RB protein binds to the transcription factor E2F keeping it in an inactive state. Mitogenic signaling causes mono-phosphorylation of RB by the cyclin D-CDK4/6 kinase in early G1 and multiple phosphorylations in late G1, which inactivates RB releasing E2F to transcribe genes for proteins required in S. p53 is continuously expressed but is kept at a low level by binding of the E3 ubiquitin ligase, MDM2, which marks it for proteasomal degradation. The cyclin C-CDK3 complex mediates the transition from G1 to G0 (quiescence). DNA damage disrupts this binding by phosphorylation of MDM2 and p53, increasing p53 levels. P53 transcriptionally activates the CDK inhibitor p21 causing transient cell cycle arrest before the G1-S transition. If the damage is not repairable, the extended G1/S arrest may be reinforced by p16, which inhibits the cyclin D-CDK4/6 complex allowing RB to bind E2F and arrest the cells permanently in G1 (premature differentiation; senescence). The activity of transcription factors and enzymes is regulated by binding proteins and phosphorylation state. Different types of protein are shown in different colors. The active and inactive forms may be either phosphorylated or unphosphorylated, depending on the specific protein. For references, see text.

Senescence of the cell is induced by long-term arrest via p53-p21 (CDKN1A) reinforced by p16 (CDKN2A) and the p14^ARF^ alternative reading frame (ARF) protein encoded by the same locus. Activation of the p16 locus contributes to p53 stabilization via ARF binding to MDM2, while p16 prevents inactivation of RB, which in turn prevents cell cycle progression and thus leads to permanent arrest [50], as illustrated in Figure 1.

**Figure 2:**
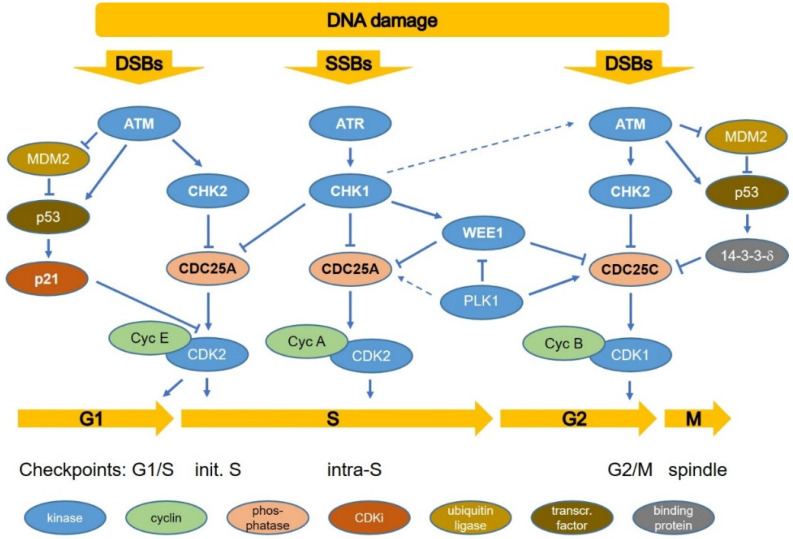
Schematic diagram of cell cycle regulation and DNA damage-induced checkpoints. The ATR kinase is activated by single-stranded DNA and stalled replication forks activating the intra-S checkpoint via CHK1 supported by WEE1. DNA double-strand breaks (DSBs) activate the ATM kinase, which mediates arrest in G1 and G2 via CHK2 supported by WEE1. Cells with wild-type p53 activate the G1/S checkpoint via p21 and contribute to the G2/M arrest. Release from G2/M arrest is mediated by increasing levels of PLK1. Different types of protein are shown in different colors (see Figure 1). The active and inactive forms may be either phosphorylated or unphosphorylated, depending on the specific protein. For further references, see the text.

Entry into S-phase from G1 is mediated by the CDK 2/cyclin E complex, which is active when dephosphorylated by CDC25A phosphatase. After initiation of the S-phase, cyclin E is replaced by cyclin A. Normal replication may be halted at stalled replication forks resulting in single-stranded regions, and if these break, single-ended DSBs are formed. RPA-coated single-stranded DNA activates the ATR-CHK1 intra-S checkpoint while DSBs activate ATM-CHK2 (Figure 2). CHK1 and CHK2 inhibit CDC25 phosphatases, thus arresting cell cycle progression. Activated ATR results in activation of CHK1 and WEE1 [34,40,44,45,46,47,51]. It was also proposed that ATR activates both CHK1 and ATM, emphasizing that the ATR-CHK1 and ATM-CHK2 do not function as distinct pathways [34].

If DSBs are detected, ATM phosphorylates CHK2, leading to the inhibition of CDC25C phosphatase activity and consequently phosphorylation and inactivation of CDK1. In the presence of SSBs, ATR phosphorylates CHK1, which activates WEE1 and inhibits the activity of CDC25C, leading to cell cycle arrest in G2 [34,40,44,45,46,47,49,52]. Radiation-induced G2/M arrest is characterized by three stages: the early (0–2 h) ATM-CHK1 response, an intermediate (2–10 h) p53 response (in *TP53* wild-type cells), and a long-term response (2–16 h and longer) with an accumulation of cells from S-phase, which is dependent on the activation of WEE1 kinase [49,53]. The G2/M block is maintained by WEE1 and MYT1, which keep CDK1 in an inactive state via phosphorylation at tyrosine 15 and threonine 14, respectively. The expression level and activity of WEE1 are associated with CHK1 activity and are increased prior to the M phase, allowing it to effectively prevent the formation and activity of the CDK1/B complex in the presence of DNA lesions. After DNA repair, the release of the G2/M block is mediated by a polo-like kinase (PLK1) with phosphorylates WEE1 (leading to its degradation) and CDC25B/C (leading to its activation), allowing the latter to dephosphorylate and thus activate CDK1.

It is important to note that all the pathways may interact at several levels. For example, the activities of ATR, ATM, and CHK1 seem to be connected via protein phosphatase 2 (PP2A) during replication stress [54]. Thus, ATM activity has been associated with ATR upon exposure to UV or during replication fork stalling [55]. Furthermore, ATM and ATR pathways are reported to play a role in mitosis [56,57,58,59].

Checkpoint inhibition may induce tumor cell death via interference with DDR, accumulation of unrepaired lesions, but also via escalation of replication stress [60,61,62]. The ATR-CHK1 pathway stabilizes replication forks and prevents their stalling, while WEE1, apart from suppressing CDK, appears to hold roles in the stability of the genome via interaction with MUS81-EME1 endonuclease and histone synthesis [63,64]. Indeed, the regulation of the cell cycle appears to be far more complex than previously known, and more studies are required for a better understanding of the mechanisms involved.

The spindle checkpoint, mediating mitotic arrest, depends on the activity of the anaphase-promoting complex (APC). The regulation of other components of APC is associated with Aurora B, which is considered crucial for the proper segregation of chromosomes. Improper chromosomal attachments activate the cell cycle arrest via inhibition of APC, which leads to the accumulation of securin, ultimately leading to the inhibition of separase, the protease which targets cohesion. This protein complex holds chromatid strands together after replication. Defects in the mitotic checkpoint inevitably result in chromosomal aberrations in the daughter cells [65,66]. Notably, CHK1 regulates the activity of Aurora B [58,67].

## 5. Inhibitors of Checkpoint Control

Inhibiting one or more cell cycle checkpoints may cause cells carrying unrepaired DNA to divide, leading to increased genomic instability and cell death [34,68]. Thus, targeting checkpoint molecules may increase the sensitivity of tumor cells to radiation- and chemotherapy. Importantly, the inherent checkpoint control dysfunctionality of tumor cells may be exploited in treatment by targeting the remaining functional control pathways in an approach called synthetic lethality. For example, G1 phase arrest and permanent G0 cell cycle arrest mainly depend on intact p53 and p16/RB pathways, which are often dysfunctional in GBM. Thus, nearly 80% of GBM carry mutations in the RB1 signaling pathway, while defects in the TP53 pathway are reported in approximately 85% of all GBM [69,70]. Therefore, combining RT with inhibitors of enzymes involved in S, G2/M, or M phase checkpoints may increase genomic instability beyond the ability of tumor cells to survive. As the intermediate and long-term cell cycle arrests in the G2/M phase are dependent on functional p53 and WEE1 kinase, the latter emerges as a particularly attractive molecular target for inhibition in combination with RT.

The development of kinase inhibitors faces certain challenges. The first is their selectivity. For example, as many inhibitors target ATP-binding sites, they could affect kinases other than the target and affect distinct pathways within cells. On the other hand, inhibiting multiple kinases involved in the targeted process may enhance the efficiency of the treatment. Another issue is the potential effect of the kinase inhibition on interconnected pathways per se. Molecules aimed to treat brain tumors face an additional challenge in the form of the blood–brain barrier. Finally, the successful inhibition of the targeted kinase in tumor cells must outweigh possible adverse effects on the normal tissue. While some kinase inhibitors have been approved for clinical practice, much work remains to be done for better understanding and efficient manipulation of cellular processes. In the following, the current knowledge of the effect of inhibition of ATM, ATR, CHK1, CHK2, and WEE1 kinases regarding the radiation treatment of brain tumors will be presented. The preclinical studies addressed in this review are summarized in Table 1.

## 6. Preclinical Studies

### 6.1. ATM and ATR

ATM is a 350,687 Da [95] DNA- and ATP-binding serine/threonine kinase that belongs to the PI3K-related protein kinase (PIKK) protein family involved in the regulation of genomic integrity, metabolism, and transcriptional regulation. Although associated with DSB repair and glucose homeostasis [96], ATM is not an essential enzyme. However, individuals carrying mutated ATM suffer from the ataxia-telangiectasia syndrome, which is characterized by neurologic movement disorder (ataxia), dilated small blood vessels (telangiectasia), high sensitivity to ionizing (but not UV) radiation, and increased risk of developing cancer [97].

Silencing the ATM gene in GSC caused radiosensitization and decreased postirradiation expression levels of p53, proliferating cell nuclear antigen (PCNA), and survivin in a mouse model and was associated with hemorrhages and necrosis in the irradiated tumors [98]. The ATM inhibitor KU-60019 successfully radiosensitized glioma cell lines, GBM-initiating cells, as well as pediatric high-grade gliomas [77,78,79]. The ability of KU-60019 to sensitize tumor cells to radiation was associated with p53 expression. The ATM inhibitor was able to radiosensitize GSC and xenografts, prolonging survival with significantly greater efficacy in p53-mutated glioma [77]. In GBM-initiating cells, the combination of ATM inhibition and RT led to the elimination of cells expressing low levels of p53 and high levels of PI3K [79]. Synthetic lethality in the context of radiosensitization was also demonstrated in p53-mut glioma cell lines, or cell lines with other checkpoint defects, which were sensitized to radiation by ATM inhibition, in contrast to wild-type p53 lines, which showed less effect of the inhibitor [81]. Notably, no signs of toxicity of KU-60019 were observed in healthy animals [80].

ATM inhibition by KU-55933 leads to radiosensitization of GBM stem cells and GBM cells and reduced G2/M checkpoint arrest and DDR, underlining the role of these processes in the phenomenon of brain tumor radioresistance [72]. On the other hand, ATM inhibition sensitized GSC to radiation, but differentiation of glioma cells resulted in the loss of the sensitizing effect [73]. In a different study, inactivation of the ATM cofactor, Atmin had a protective role against GBM formation, resulting in sensitization to hypoxia and decreased level of platelet-derived growth factor receptor alpha (PDGFRA) expression in mice carrying *TP53* mutations. Inhibition of ATM reduced the expression of PDGFRA and proliferation of primary GBM stem culture while not affecting normal neural stem cells [74]. In a study on glioma-initiating cells comparing the effect of KU55933 with an abrogation of NHEJ by a DNA-dependent protein kinase inhibitor (DNA-PKi), the combination of ATM inhibition and RT prolonged the survival of tumor-bearing mice, suggesting that HR dominates the survival of these cells [75].

Besides interfering with DSB repair, ATM inhibition may indirectly confer radiosensitivity. Thus, inhibition of ATM counteracted the pro-survival effect of interleukin-1 (IL-1) in GBM cells, presumably by preventing transcription and activation of NF-kB [99]. ATM was also associated with immune and metabolic modulators in the response of glioma cells to radiation. Inhibition of ATM abolished the activities of NFκB and TP53-induced glycolysis and apoptosis regulator (TIGAR), resulting in reduced cytotoxicity of TNFα and the radiomimetic Neocarzinostatin (NCS) [100].

An association of ATM with the sensitivity of glioma cells to TMZ has been reported, which may be relevant for the combination with RT. Thus, inhibition of ATM sensitized TMZ-sensitive but notresistant glioma cell lines to treatment with TMZ [101].

Inhibition of ATR in the context of irradiating brain tumors has been less well studied. ATR is a 301,367 Da [102] DNA- and ATP-binding serine/threonine kinase and is also a member of the PIKK protein family involved in maintaining genomic integrity. The activity of this enzyme depends on its complexing with ATRIP and the persistent presence of SSB, while its well-known substrate is CHK1. ATR is essential for survival, and ATR deficiency is embryonic lethal [103]. As a checkpoint for replication stress and DNA damage in S-phase, ATR is a potential target for sensitizing tumor cells to DNA damage, but the cells’ response to ATR inhibition may depend on the functionality of the DDR machinery [104]. DNA synthesis was reported to be slower and permeated with a higher number of stalled replication forks in CD133+ GSC in comparison with non-GSC GBM cells [83]. A higher number of DSBs was observed in GSC during DNA replication, particularly in the locations of the replication machinery, fragile sites, and DNA:RNA hybrids, and it was hypothesized that radioresistance of GBM GSCs may be caused by continuous activation of DDR by replication stress. Combined inhibition of ATR and PARP, an enzyme involved in the stabilization of replication forks, resulted in a significant reduction of resistance to radiation in both GSC and tumor bulk populations. However, the resulting number of DSBs was higher in GSCs, marking them as particularly sensitive to impairment of the S phase checkpoint [83]. Despite these promising results, NVP-BEZ235, a broad-spectrum inhibitor of PI3K, mTOR and ATR, and AZD6738, an ATR-specific inhibitor, failed to prolong the survival of mice bearing primary GSC-derived tumors when used alone or together in combination with RT, compared with the effect of radiation alone [82].

Combined inhibition of multiple targets may enhance the effectiveness of tumor treatment. Inhibition of ATR and poly (ADP-ribose) polymerase (PARP) showed a synergistic effect on the viability of GBM cells, as well as on the survival of the treated animals carrying orthotopic brain tumors [84]. As an alternative to using multiple inhibitors, a single nonspecific inhibitor may be used for targeting multiple kinases. An example of such an inhibitor used to treat brain tumor cells is NVP-BEZ235, an inhibitor of multiple targets that include ATM and DNA-PK catalytic subunit (DNA-PKcs), which was associated with the increased sensitivity of glioma stem cell xerographs in mice to RT and TMZ [71,105]. However, in the absence of evidence of genetic targeting of *ATR* expression, the results provided by inhibitors targeting multiple pathways should be interpreted with caution.

In addition to its primary function associated with SSBs, ATR is also involved in alternative lengthening of telomeres (ALT), a mechanism carried out by the HR machinery, in which cells of many tumor types restore the telomeres achieve immortality. Thus, inhibition of ATR was linked with the prevention of ALT and induction of cell death in ALT-positive tumors [106,107].

A recent study has associated sensitivity to ATR inhibition with the MGMT status in the GBM cell line LN229, characterized by MGMT promoter methylation, which was sensitive to TMZ and ATR inhibition in contrast with cells carrying an MGMT open reading frame [108].

### 6.2. CHK1 and CHK2

CHK1 and CHK2 are ATP-binding serine/threonine protein kinases that are involved in several steps of the cell cycle progression and hence the maintenance of genomic integrity. CHK1 is a 54,434 Da [109] protein activated by ATR, and its targets include the phosphatases CDC25A, CDC25B, and CDC25C involved in regulating the S and G1 cell cycle phase. CHK2, with a molecular weight of 60,915 Da [110], is a substrate of ATM and, therefore primarily involved in the cellular response to DSBs. While the consequences of CHK1 inhibition have been addressed over the past two decades, the inhibition of CHK2 remains less well studied.

Debromohymenialdisine (DBH), an inhibitor of CHK1 and CHK2 kinases, was shown to have a synergistic toxic effect with RT on CD133-expressing GSC, both in vitro and in an animal model [38]. While CHK1 gene knockdown successfully decreased radioresistance of GSC with reduced G2/M arrest and increased apoptosis, this was not the case with the inhibition of CHK2 [111]. A similar effect was observed in studies of colon and pancreatic tumors [112,113]. While these reports seem to favor the inhibition of CHK1 over CHK2, the inhibition of CHK2 successfully increased the radiosensitivity of meningioma cells [90].

Several studies demonstrated the importance of targeting multiple kinases, such as the combination of ATM and PARP [76] and the synergistic killing effect of GBM cells by combining CHK1 and MEK1/2 inhibition [86]. Similarly, CHK1 inhibition combined with RT, TMZ, and the DNA-hypomethylating drug decitabine showed a stronger antiproliferative effect compared with individual treatments [89].

The inhibition of CHK1 by UCN-01 had a moderately radiosensitizing effect on GBM cells, leaving them with a large number of unrepaired DSBs, whereas transfer of conditioned medium increased the survival of unirradiated bystander cells [87]. UCN-01 reduced the growth of glioma cells and increased their sensitivity to TMZ, cisplatin, and 1,3-bis(2-chloroethyl)-1-nitrosourea (BCNU) treatments [114]. The effectiveness of UCN-01 was associated with the inhibition of CHK1, phosphoinositide-dependent kinase 1 (PDK1), and reduced protein kinase C (PKC) activity [115]. Additionally, it was shown that successful induction of apoptosis depends on the duration of UCN-01 exposure [85].

The inhibition of CHK1 and ATR were shown to be cytotoxic in c-MYC overexpressing medulloblastoma cells. Interestingly, while the inhibition of CHK1 sensitized medulloblastoma cells to cisplatin, it had no effect on their radiosensitivity [116].

UCN-01 abrogated G2/M cell cycle arrest and enhanced the cytotoxic effect of TMZ independently of p53 status [117], whereas inhibition of CHK2 activity was associated with suppression of TMZ efficacy in AKT-overexpressing GBM cells [118].

### 6.3. WEE1

WEE1 is a 71,597 Da [116] ATP-binding serine/threonine protein kinase that plays a crucial role in G2 cell cycle arrest and, therefore, in genome maintenance. As the inhibition of WEE1 activity enables the cell to divide in the presence of DNA abnormalities, the combination of WEE1 inhibition and RT may drive cells into mitotic catastrophe.

Mir et al. [88] demonstrated a role of WEE1 kinase in genomic stability of GBM and a significant effect of combining WEE1 inhibition and irradiation on the viability of established GBM cell lines, GBM stem-like cells, primary GBM cultures, and animal models. WEE1 kinase was overexpressed in GBM patients, and the WEE1 expression level was shown to correlate with patients’ survival. The WEE1 inhibitor PD0166285 in combination with RT reduced the viability of GBM cells, reduced tumor burden, and prolonged animal survival. In addition, inhibition of CHK1 with UCN-01 had a radiosensitizing effect on GBM, reducing their mitotic potential and leaving them with a large number of DSBs. It should be noted that this study also included expression profiles of the kinases in different tumor types showing that the response to the kinase-base treatment may depend on the type of the tumor [88]. In a similar study, WEE1 inhibition prevented the accumulation of irradiated cells in the G2 phase, however, this effect was temporary for the GBM stem cell lines [91]. While the MK-1775 inhibitor sensitized both p53-wt and p53-mutated cell lines to radiation, it failed to radiosensitize the G179 line, which was characterized by high expression of WEE1. On the other hand, an enhanced response to the WEE1 inhibitor MK-1775 in combination with RT was observed after knockdown or knockout of p21 in tumor and normal cell lines [119]. This was associated with an increased number of DNA lesions marked by γH2AX in the S-phase and suggested that the efficacy of WEE1 inhibition might depend on p53 status in some settings. In the in vivo study on glioblastoma, a synergistic effect of the combination of MK-1775 with fractionated RT on tumor growth delay was observed, which was associated with an enhanced mitotic rate [91]. Another study involving MK-1775 demonstrated the ability of the inhibitor to radiosensitize GBM in vitro, but failure to synergize with TMZ in vivo was ascribed to the limited access to the target tumor cells [92].

WEE1 was found to be highly expressed in diffuse intrinsic pontine glioma (DIPG; now known as diffuse midline glioma) and in pediatric high-grade gliomas (HGGs) [93,94]. The inhibition of WEE1 activity increased the radiosensitivity of DIPG cells in vitro, reduced tumor burden in vivo, and prolonged the survival of orthotopic mice. In HGGs, higher WEE1 expression levels were associated with higher tumor grades. Inhibition of WEE1 radiosensitized HGG cells, when applied 24 h after the RT, decreased tumor burden and increased survival in RT-treated animals.

The potential role of cell cycle checkpoint inhibition in the treatment of high-grade glioma was supported by the dose-dependent radiosensitizing effect of MEK162, the MAPK-targeting agent, which downregulated the expression of WEE1, ATM, and CHK2 kinase, as well as CDK1 and CDK2. The synergistic effect of combined MEK162-irradiation on tumor growth was observed in a primary GBM orthotopic model, while combined treatment and the inhibitor alone prolonged survival [120].

A recent study on non-brain murine tumors showed that, in addition to sensitization of radiation-induced tumor cell inactivation, inhibition of the WEE1 kinase also enhanced the response to immune checkpoint blockade [121]. This potentially important finding suggests that radiation-induced mitotic catastrophe may release DAMP molecules and possibly neoantigens to stimulate the antitumor immune response [122,123].

## 7. Clinical Studies

Several clinical phase I-II trials of checkpoint kinase inhibitors in combination with RT and CT in solid tumors or leukemia have been published or are ongoing, but a comprehensive review included only two ongoing trials on brain tumors [124]. A recent review discussed molecular targets in the treatment of high-grade pediatric glioma, but no clinical studies on cell cycle checkpoint kinase inhibitors were listed [125].

A search of the NIH ClinicalTrials.gov database [126] for clinical trials on cell cycle checkpoint inhibitors in brain tumors identified two trials on inhibitors of ATM and CHK1, respectively, and five on the WEE1 inhibitor AZD1775 (Table 2). Notably, three ongoing phase I trials are testing the combination of AZD1775 (Adavosertib) with RT. One phase 0/early phase I trial (NCT02207010) published results showing good brain tumor penetration, which contrasted an earlier preclinical study showing limited uptake [92,127]. Since RT increases the blood–brain barrier permeability, inhibitor uptake may be further enhanced by combination therapy [128]. Clearly, there is a need for more trials on inhibitor-plus-RT combinations in well-characterized primary brain tumors, including the identification of biomarkers for sensitivity and resistance.

## 8. Conclusions

RT is applied as a standard of care treatment for many brain tumors. Nevertheless, the effectiveness of RT is limited by the radioresistance of tumor tissue, particularly tumor stem cells. The application of RT is restricted by the level of tolerance of the normal tissue, thus, the prognosis for the most aggressive brain tumors has remained very poor over the past decades. Overall, the treatment of brain tumors needs advancement, and the abrogation of mechanisms involved in the radioresistance of tumor cells seems a promising avenue for further investigation. Cell cycle checkpoint inhibition is still in its infancy but while ATM, CHK1, and WEE1 inhibitors resulted in successful sensitization of various tumor types, including primary brain tumors, ATM and CHK2 inhibitors are less studied in brain tumors.

The preclinical studies strongly suggest that cellular and genetic context is an important factor in determining the radiosensitizing effect of different kinase inhibitors. This suggestion implies a need for reliable biomarkers to predict which tumors are likely to respond to specific inhibitors or specific combinations of different inhibitors. For example, *TP53* status seems to be important for the efficacy of some inhibitors, and current data agree with the notion of synthetic lethality of ATM inhibition in cells with a p53-defective pathway when combined with irradiation. Overall, the preclinical data support the hypothesis that cell cycle checkpoint kinases may be clinically valuable targets in brain tumors and encourage further clinical trials in that area. It should be noted that the effect of the inhibition treatment depends not only on cell types but also on the selectivity and pharmacokinetics of the applied inhibitor. Furthermore, the mechanisms regulating the cell cycle still need to be fully illuminated in order to identify the best therapeutic target(s). A promising option is the potential enhancement of antitumor immune response by combining cell cycle kinase inhibitors with RT and immune checkpoint inhibitors.

## Figures and Tables

**Table 1:** Kinases targeted by inhibitors in combination with RT in brain tumors.

Protein Name (*Gene Symbol*)	Molecular Weight	Molecular Function	Inhibitor Used for Addressed Target in Combination with RT on Brain Tumor Models	References
ATM (*ATM*)	350,687 Da	DNA- and ATP-binding Serine/threonine protein kinase	NVP-BEZ235	[71]
			KU-55933	[72,73,74,75,76]
			KU-60019	[76,77,78,79]
			AZD1390	[80]
			AZ31 and AZ32	[81]
ATR (*ATR*)	301,367 Da	DNA- and ATP-binding Serine/threonine protein kinase	NVP-BEZ235	[82]
			AZD6738	[82]
			VE821	[83,84,85]
			VE822	[84]
			AZ20	[84]
CHK1 (*CHEK1*)	54,434 Da	ATP-binding Serine/threonine protein kinase	Gö-6976	[73]
			UCN-01	[86,87,88]
			debromohymenialdisine	[38]
			SAR-020106	[89]
			SCH900776	[76]
			CHIR-124	[76]
CHK2 (*CHEK2*)	60,915 Da	ATP-binding Serine/threonine protein kinase	BML-277	[73]
			debromohymenialdisine	[38]
			NSC 109555	[90]
WEE1 (*WEE1*)	71,597 Da	ATP-binding Serine/threonine protein kinase	MK-1775	[80,91,92,93,94]
			PD0166285	[88]

Studies based solely on gene knockdown or RNA interference are not included in the table.

**Table 2:** Clinical trials involving inhibitors of ATM, CHK1, and WEE1 in brain tumors [126].

Target	Drug	Phase	Estimated End Date/End Date	Tumor	ClinicalTrials.Gov Identifier	References	Status	Other Treatments
ATM	AZD1390	I	14 December 2023	Brain tumors, Leptomeningeal disease	NCT03423628	[129]	Active	RT
CHK1	LY2606368	I	June 2026	Medulloblastoma in pediatric patients	NCT04023669	[130]	Active	Cyclophosphamide Gemcitabine Filgrastim peg-filgrastim
WEE1	AZD1775 (MK-1775)	I	25 March 2019	Recurrent glioblastoma	NCT02207010	[127,131]	Completed	RT
WEE1	AZD1775 (MK-1775)	I	29 July 2021, primary	Glioblastoma	NCT01849146	[132]	Active	RT, TMZ
WEE1	AZD1775 (MK-1775)	I	31 October 2021, primary	Diffuse intrinsic pontine gliomas	NCT01922076	[133]	Active	RT
WEE1	AZD1775 (MK-1775)	I	31 December 2021	Relapsed or refractory solid tumors (including medulloblastoma)	NCT02095132	[134]	Active	Irinotecan hydrochloride
WEE1	AZD1775 (MK-1775)	II	30 June 2022	Advanced refractory solid tumors (including gliomas), lymphomas, or multiple myeloma	NCT02465060	[135]	Active	Multiple drugs

## References

[1] McFaline-Figueroa J.R., Lee E.Q. (2018). Brain Tumors. Am. J. Med..

[2] Ostrom Q.T., Cioffi G., Gittleman H., Patil N., Waite K., Kruchko C., Barnholtz-Sloan J.S. (2019). CBTRUS Statistical Report: Primary Brain and Other Central Nervous System Tumors Diagnosed in the United States in 2012–2016. Neuro Oncol..

[3] Udaka Y.T., Packer R.J. (2018). Pediatric Brain Tumors. Neurol. Clin..

[4] Stupp R., Mason W.P., van den Bent M.J., Weller M., Fisher B., Taphoorn M.J.B., Belanger K., Brandes A.A., Marosi C., Bogdahn U. (2005). Radiotherapy plus Concomitant and Adjuvant Temozolomide for Glioblastoma. N. Engl. J. Med..

[5] Marchesi F., Turriziani M., Tortorelli G., Avvisati G., Torino F., De Vecchis L. (2007). Triazene compounds: Mechanism of action and related DNA repair systems. Pharmacol. Res..

[6] Stupp R., Hegi M.E., Mason W.P., van den Bent M.J., Taphoorn M.J.B., Janzer R.C., Ludwin S.K., Allgeier A., Fisher B., Belanger K. (2009). Effects of radiotherapy with concomitant and adjuvant temozolomide versus radiotherapy alone on survival in glioblastoma in a randomised phase III study: 5-year analysis of the EORTC-NCIC trial. Lancet Oncol..

[7] Weller M., van den Bent M., Tonn J.C., Stupp R., Preusser M., Cohen-Jonathan-Moyal E., Henriksson R., Le Rhun E., Balana C., Chinot O. (2017). European Association for Neuro-Oncology (EANO) guideline on the diagnosis and treatment of adult astrocytic and oligodendroglial gliomas. Lancet Oncol..

[8] Lomax M.E., Folkes L.K., O’Neill P. (2013). Biological consequences of radiation-induced DNA damage: Relevance to radiotherapy. Clin. Oncol. (R. Coll. Radiol.).

[9] Iliakis G., Mladenov E., Mladenova V. (2019). Necessities in the Processing of DNA Double Strand Breaks and Their Effects on Genomic Instability and Cancer. Cancers.

[10] Kaina B., Christmann M. (2019). DNA repair in personalized brain cancer therapy with temozolomide and nitrosoureas. DNA Repair.

[11] Elmore K.B., Schaff L.R. (2021). DNA Repair Mechanisms and Therapeutic Targets in Glioma. Curr. Oncol. Rep..

[12] Ou A., Yung W.K.A., Majd N. (2021). Molecular Mechanisms of Treatment Resistance in Glioblastoma. Int. J. Mol. Sci..

[13] Hanahan D., Weinberg R.A. (2011). Hallmarks of cancer: The next generation. Cell.

[14] Lee E., Yong R.L., Paddison P., Zhu J. (2018). Comparison of glioblastoma (GBM) molecular classification methods. Semin. Cancer Biol..

[15] Phillips H.S., Kharbanda S., Chen R., Forrest W.F., Soriano R.H., Wu T.D., Misra A., Nigro J.M., Colman H., Soroceanu L. (2006). Molecular subclasses of high-grade glioma predict prognosis, delineate a pattern of disease progression, and resemble stages in neurogenesis. Cancer Cell.

[16] Verhaak R.G., Hoadley K.A., Purdom E., Wang V., Qi Y., Wilkerson M.D., Miller C.R., Ding L., Golub T., Mesirov J.P. (2010). Integrated genomic analysis identifies clinically relevant subtypes of glioblastoma characterized by abnormalities in PDGFRA, IDH1, EGFR, and NF1. Cancer Cell.

[17] Wang Q., Hu B., Hu X., Kim H., Squatrito M., Scarpace L., de Carvalho A.C., Lyu S., Li P., Li Y. (2017). Tumor Evolution of Glioma-Intrinsic Gene Expression Subtypes Associates with Immunological Changes in the Microenvironment. Cancer Cell.

[18] Patel A.P., Tirosh I., Trombetta J.J., Shalek A.K., Gillespie S.M., Wakimoto H., Cahill D.P., Nahed B.V., Curry W.T., Martuza R.L. (2014). Single-cell RNA-seq highlights intratumoral heterogeneity in primary glioblastoma. Science.

[19] Neftel C., Laffy J., Filbin M.G., Hara T., Shore M.E., Rahme G.J., Richman A.R., Silverbush D., Shaw M.L., Hebert C.M. (2019). An Integrative Model of Cellular States, Plasticity, and Genetics for Glioblastoma. Cell.

[20] Calabrese C., Poppleton H., Kocak M., Hogg T.L., Fuller C., Hamner B., Oh E.Y., Gaber M.W., Finklestein D., Allen M. (2007). A perivascular niche for brain tumor stem cells. Cancer Cell.

[21] Arnold C.R., Mangesius J., Skvortsova I.-I., Ganswindt U. (2020). The Role of Cancer Stem Cells in Radiation Resistance. Front. Oncol..

[22] Beck B., Blanpain C. (2013). Unravelling cancer stem cell potential. Nat. Rev. Cancer.

[23] Wang Y., Xu H., Liu T., Huang M., Butter P.P., Li C., Zhang L., Kao G.D., Gong Y., Maity A. (2018). Temporal DNA-PK activation drives genomic instability and therapy resistance in glioma stem cells. JCI Insight.

[24] Lim Y.C., Roberts T.L., Day B.W., Harding A., Kozlov S., Kijas A.W., Ensbey K.S., Walker D.G., Lavin M.F. (2012). A role for homologous recombination and abnormal cell-cycle progression in radioresistance of glioma-initiating cells. Mol. Cancer.

[25] Morrison C., Weterings E., Mahadevan D., Sanan A., Weinand M., Stea B. (2021). Expression Levels of RAD51 Inversely Correlate with Survival of Glioblastoma Patients. Cancers.

[26] Narayan R.S., Fedrigo C.A., Stalpers L.J., Baumert B.G., Sminia P. (2013). Targeting the Akt-pathway to improve radiosensitivity in glioblastoma. Curr. Pharm. Des..

[27] Kumar V., Vashishta M., Kong L., Wu X., Lu J.J., Guha C., Dwarakanath B.S. (2021). The Role of Notch, Hedgehog, and Wnt Signaling Pathways in the Resistance of Tumors to Anticancer Therapies. Front. Cell Dev. Biol..

[28] Aldaz P., Arozarena I. (2021). Tyrosine Kinase Inhibitors in Adult Glioblastoma: An (Un)Closed Chapter?. Cancers.

[29] Schafer N., Gielen G.H., Rauschenbach L., Kebir S., Till A., Reinartz R., Simon M., Niehusmann P., Kleinschnitz C., Herrlinger U. (2019). Longitudinal heterogeneity in glioblastoma: Moving targets in recurrent versus primary tumors. J. Transl. Med..

[30] Van den Bent M.J., Gao Y., Kerkhof M., Kros J.M., Gorlia T., van Zwieten K., Prince J., van Duinen S., Sillevis Smitt P.A., Taphoorn M. (2015). Changes in the EGFR amplification and EGFRvIII expression between paired primary and recurrent glioblastomas. Neuro Oncol..

[31] Chakravarti A., Loeffler J.S., Dyson N.J. (2002). Insulin-like growth factor receptor I mediates resistance to anti-epidermal growth factor receptor therapy in primary human glioblastoma cells through continued activation of phosphoinositide 3-kinase signaling. Cancer Res..

[32] Wang M., Maier P., Wenz F., Giordano F.A., Herskind C. (2013). Mitogenic signalling in the absence of epidermal growth factor receptor activation in a human glioblastoma cell line. J. NeuroOncol..

[33] Lauko A., Lo A., Ahluwalia M.S., Lathia J.D. (2021). Cancer cell heterogeneity & plasticity in glioblastoma and brain tumors. Semin. Cancer Biol..

[34] Nakanishi M., Shimada M., Niida H. (2006). Genetic instability in cancer cells by impaired cell cycle checkpoints. Cancer Sci..

[35] Morgan M.A., Lawrence T.S. (2015). Molecular Pathways: Overcoming Radiation Resistance by Targeting DNA Damage Response Pathways. Clin. Cancer Res..

[36] Harris S.L., Levine A.J. (2005). The p53 pathway: Positive and negative feedback loops. Oncogene.

[37] Blackford A.N., Jackson S.P. (2017). ATM, ATR, and DNA-PK: The Trinity at the Heart of the DNA Damage Response. Mol. Cell.

[38] Bao S., Wu Q., McLendon R.E., Hao Y., Shi Q., Hjelmeland A.B., Dewhirst M.W., Bigner D.D., Rich J.N. (2006). Glioma stem cells promote radioresistance by preferential activation of the DNA damage response. Nature.

[39] De Bacco F., D’Ambrosio A., Casanova E., Orzan F., Neggia R., Albano R., Verginelli F., Cominelli M., Poliani P.L., Luraghi P. (2016). MET inhibition overcomes radiation resistance of glioblastoma stem-like cells. EMBO Mol. Med..

[40] Barnum K.J., O’Connell M.J. (2014). Cell cycle regulation by checkpoints. Methods Mol. Biol..

[41] Fischer M., Muller G.A. (2017). Cell cycle transcription control: DREAM/MuvB and RB-E2F complexes. Crit. Rev. Biochem. Mol. Biol..

[42] Bertoli C., Skotheim J.M., de Bruin R.A. (2013). Control of cell cycle transcription during G1 and S phases. Nat. Rev. Mol. Cell Biol..

[43] Narasimha A.M., Kaulich M., Shapiro G.S., Choi Y.J., Sicinski P., Dowdy S.F. (2014). Cyclin D activates the Rb tumor suppressor by mono-phosphorylation. eLife.

[44] Campos A., Clemente-Blanco A. (2020). Cell Cycle and DNA Repair Regulation in the Damage Response: Protein Phosphatases Take Over the Reins. Int. J. Mol. Sci..

[45] Ding L., Cao J., Lin W., Chen H., Xiong X., Ao H., Yu M., Lin J., Cui Q. (2020). The Roles of Cyclin-Dependent Kinases in Cell-Cycle Progression and Therapeutic Strategies in Human Breast Cancer. Int. J. Mol. Sci..

[46] Kastan M.B., Bartek J. (2004). Cell-cycle checkpoints and cancer. Nature.

[47] Latif C., Harvey S.H., O’Connell M.J. (2001). Ensuring the stability of the genome: DNA damage checkpoints. Sci. World J..

[48] Ren S., Rollins B.J. (2004). Cyclin C/cdk3 promotes Rb-dependent G0 exit. Cell.

[49] Deckbar D., Jeggo P.A., Lobrich M. (2011). Understanding the limitations of radiation-induced cell cycle checkpoints. Crit. Rev. Biochem. Mol. Biol..

[50] Campisi J., d’Adda di Fagagna F. (2007). Cellular senescence: When bad things happen to good cells. Nat. Rev. Mol. Cell Biol..

[51] Sowd G.A., Li N.Y., Fanning E. (2013). ATM and ATR activities maintain replication fork integrity during SV40 chromatin replication. PLoS Pathog..

[52] Matheson C.J., Backos D.S., Reigan P. (2016). Targeting WEE1 Kinase in Cancer. Trends Pharm. Sci..

[53] Landsverk K.S., Patzke S., Rein I.D., Stokke C., Lyng H., De Angelis P.M., Stokke T. (2011). Three independent mechanisms for arrest in G2 after ionizing radiation. Cell Cycle.

[54] Palii S.S., Cui Y., Innes C.L., Paules R.S. (2013). Dissecting cellular responses to irradiation via targeted disruptions of the ATM-CHK1-PP2A circuit. Cell Cycle.

[55] Stiff T., Walker S.A., Cerosaletti K., Goodarzi A.A., Petermann E., Concannon P., O’Driscoll M., Jeggo P.A. (2006). ATR-dependent phosphorylation and activation of ATM in response to UV treatment or replication fork stalling. EMBO J..

[56] Agarwal M.L., Agarwal A., Taylor W.R., Stark G.R. (1995). p53 controls both the G2/M and the G1 cell cycle checkpoints and mediates reversible growth arrest in human fibroblasts. Proc. Natl. Acad. Sci. USA.

[57] Eliezer Y., Argaman L., Kornowski M., Roniger M., Goldberg M. (2014). Interplay between the DNA damage proteins MDC1 and ATM in the regulation of the spindle assembly checkpoint. J. Biol. Chem..

[58] Mackay D.R., Ullman K.S. (2015). ATR and a Chk1-Aurora B pathway coordinate postmitotic genome surveillance with cytokinetic abscission. Mol. Biol. Cell.

[59] Smith E., Dejsuphong D., Balestrini A., Hampel M., Lenz C., Takeda S., Vindigni A., Costanzo V. (2009). An ATM- and ATR-dependent checkpoInt. inactivates spindle assembly by targeting CEP63. Nat. Cell Biol..

[60] Mustofa M.K., Tanoue Y., Tateishi C., Vaziri C., Tateishi S. (2020). Roles of Chk2/CHEK2 in guarding against environmentally induced DNA damage and replication-stress. Environ. Mol. Mutagenesis.

[61] Ngoi N.Y.L., Pham M.M., Tan D.S.P., Yap T.A. (2021). Targeting the replication stress response through synthetic lethal strategies in cancer medicine. Trends Cancer.

[62] Zhang J., Dai Q., Park D., Deng X. (2016). Targeting DNA Replication Stress for Cancer Therapy. Genes.

[63] Dominguez-Kelly R., Martin Y., Koundrioukoff S., Tanenbaum M.E., Smits V.A., Medema R.H., Debatisse M., Freire R. (2011). Wee1 controls genomic stability during replication by regulating the Mus81-Eme1 endonuclease. J. Cell Biol..

[64] Mahajan K., Mahajan N.P. (2013). WEE1 tyrosine kinase, a novel epigenetic modifier. Trends Genet..

[65] Bharadwaj R., Yu H. (2004). The spindle checkpoint, aneuploidy, and cancer. Oncogene.

[66] Sasai K., Treekitkarnmongkol W., Kai K., Katayama H., Sen S. (2016). Functional Significance of Aurora Kinases-p53 Protein Family Interactions in Cancer. Front. Oncol..

[67] Kabeche L., Nguyen H.D., Buisson R., Zou L. (2018). A mitosis-specific and R loop-driven ATR pathway promotes faithful chromosome segregation. Science.

[68] Spoerri L., Oo Z.Y., Larsen J.E., Haass N.K., Gabrielli B., Pavey S., Wondrak G.T. (2015). Cell Cycle CheckpoInt. and DNA Damage Response Defects as Anticancer Targets: From Molecular Mechanisms to Therapeutic Opportunities. Stress Response Pathways in Cancer: From Molecular Targets to Novel Therapeutics.

[69] Pearson J.R.D., Regad T. (2017). Targeting cellular pathways in glioblastoma multiforme. Signal. Transduct Target..

[70] Zhang Y., Dube C., Gibert M., Cruickshanks N., Wang B., Coughlan M., Yang Y., Setiady I., Deveau C., Saoud K. (2018). The p53 Pathway in Glioblastoma. Cancers.

[71] Gil del Alcazar C.R., Hardebeck M.C., Mukherjee B., Tomimatsu N., Gao X., Yan J., Xie X.J., Bachoo R., Li L., Habib A.A. (2014). Inhibition of DNA double-strand break repair by the dual PI3K/mTOR inhibitor NVP-BEZ235 as a strategy for radiosensitization of glioblastoma. Clin. Cancer Res..

[72] Carruthers R., Ahmed S.U., Strathdee K., Gomez-Roman N., Amoah-Buahin E., Watts C., Chalmers A.J. (2015). Abrogation of radioresistance in glioblastoma stem-like cells by inhibition of ATM kinase. Mol. Oncol..

[73] Raso A., Vecchio D., Cappelli E., Ropolo M., Poggi A., Nozza P., Biassoni R., Mascelli S., Capra V., Kalfas F. (2012). Characterization of glioma stem cells through multiple stem cell markers and their specific sensitization to double-strand break-inducing agents by pharmacological inhibition of ataxia telangiectasia mutated protein. Brain Pathol.

[74] Blake S.M., Stricker S.H., Halavach H., Poetsch A.R., Cresswell G., Kelly G., Kanu N., Marino S., Luscombe N.M., Pollard S.M. (2016). Inactivation of the ATMIN/ATM pathway protects against glioblastoma formation. eLife.

[75] Lim Y.C., Roberts T.L., Day B.W., Stringer B.W., Kozlov S., Fazry S., Bruce Z.C., Ensbey K.S., Walker D.G., Boyd A.W. (2014). Increased sensitivity to ionizing radiation by targeting the homologous recombination pathway in glioma initiating cells. Mol. Oncol..

[76] Ahmed S.U., Carruthers R., Gilmour L., Yildirim S., Watts C., Chalmers A.J. (2015). Selective Inhibition of Parallel DNA Damage Response Pathways Optimizes Radiosensitization of Glioblastoma Stem-like Cells. Cancer Res..

[77] Biddlestone-Thorpe L., Sajjad M., Rosenberg E., Beckta J.M., Valerie N.C., Tokarz M., Adams B.R., Wagner A.F., Khalil A., Gilfor D. (2013). ATM kinase inhibition preferentially sensitizes p53-mutant glioma to ionizing radiation. Clin. Cancer Res..

[78] Vecchio D., Daga A., Carra E., Marubbi D., Raso A., Mascelli S., Nozza P., Garre M.L., Pitto F., Ravetti J.L. (2015). Pharmacokinetics, pharmacodynamics and efficacy on pediatric tumors of the glioma radiosensitizer KU60019. Int. J. Cancer.

[79] Vecchio D., Daga A., Carra E., Marubbi D., Baio G., Neumaier C.E., Vagge S., Corvo R., Pia Brisigotti M., Louis Ravetti J. (2014). Predictability, efficacy and safety of radiosensitization of glioblastoma-initiating cells by the ATM inhibitor KU-60019. Int. J. Cancer.

[80] Durant S.T., Zheng L., Wang Y., Chen K., Zhang L., Zhang T., Yang Z., Riches L., Trinidad A.G., Pass M. (2018). The brain-penetrant clinical ATM inhibitor AZD1390 radiosensitizes and improves survival of preclinical brain tumor models. Sci. Adv..

[81] Karlin J., Allen J., Ahmad S.F., Hughes G., Sheridan V., Odedra R., Farrington P., Cadogan E.B., Riches L.C., Garcia-Trinidad A. (2018). Orally Bioavailable and Blood-Brain Barrier-Penetrating ATM Inhibitor (AZ32) Radiosensitizes Intracranial Gliomas in Mice. Mol. Cancer.

[82] Frosina G., Profumo A., Marubbi D., Marcello D., Ravetti J.L., Daga A. (2018). ATR kinase inhibitors NVP-BEZ235 and AZD6738 effectively penetrate the brain after systemic administration. Radiat. Oncol..

[83] Carruthers R.D., Ahmed S.U., Ramachandran S., Strathdee K., Kurian K.M., Hedley A., Gomez-Roman N., Kalna G., Neilson M., Gilmour L. (2018). Replication Stress Drives Constitutive Activation of the DNA Damage Response and Radioresistance in Glioblastoma Stem-like Cells. Cancer Res..

[84] Ning J.F., Stanciu M., Humphrey M.R., Gorham J., Wakimoto H., Nishihara R., Lees J., Zou L., Martuza R.L., Wakimoto H. (2019). Myc targeted CDK18 promotes ATR and homologous recombination to mediate PARP inhibitor resistance in glioblastoma. Nat. Commun..

[85] Bredel M., Pollack I.F., Freund J.M., Rusnak J., Lazo J.S. (1999). Protein kinase C inhibition by UCN-01 induces apoptosis in human glioma cells in a time-dependent fashion. J. NeuroOncol..

[86] Tang Y.Y., Grant S., Dent P. (2012). Enhancing CHK1 inhibitor lethality in glioblastoma. Cancer Biol..

[87] Burdak-Rothkamm S., Rothkamm K., McClelland K., Al Rashid S.T., Prise K.M. (2015). BRCA1, FANCD2 and Chk1 are potential molecular targets for the modulation of a radiation-induced DNA damage response in bystander cells. Cancer Lett..

[88] Mir S.E., De Witt Hamer P.C., Krawczyk P.M., Balaj L., Claes A., Niers J.M., Van Tilborg A.A., Zwinderman A.H., Geerts D., Kaspers G.J. (2010). In silico analysis of kinase expression identifies WEE1 as a gatekeeper against mitotic catastrophe in glioblastoma. Cancer Cell.

[89] Patties I., Kallendrusch S., Bohme L., Kendzia E., Oppermann H., Gaunitz F., Kortmann R.D., Glasow A. (2019). The Chk1 inhibitor SAR-020106 sensitizes human glioblastoma cells to irradiation, to temozolomide, and to decitabine treatment. J. Exp. Clin. Cancer Res..

[90] Gogineni V.R., Nalla A.K., Gupta R., Dinh D.H., Klopfenstein J.D., Rao J.S. (2011). Chk2-mediated G2/M cell cycle arrest maintains radiation resistance in malignant meningioma cells. Cancer Lett..

[91] Sarcar B., Kahali S., Prabhu A.H., Shumway S.D., Xu Y., Demuth T., Chinnaiyan P. (2011). Targeting radiation-induced G(2) checkpoInt. activation with the Wee-1 inhibitor MK-1775 in glioblastoma cell lines. Mol. Cancer.

[92] Pokorny J.L., Calligaris D., Gupta S.K., Iyekegbe D.O., Mueller D., Bakken K.K., Carlson B.L., Schroeder M.A., Evans D.L., Lou Z. (2015). The Efficacy of the Wee1 Inhibitor MK-1775 Combined with Temozolomide Is Limited by Heterogeneous Distribution across the Blood-Brain Barrier in Glioblastoma. Clin. Cancer Res..

[93] Mueller S., Hashizume R., Yang X., Kolkowitz I., Olow A.K., Phillips J., Smirnov I., Tom M.W., Prados M.D., James C.D. (2014). Targeting Wee1 for the treatment of pediatric high-grade gliomas. Neuro Oncol..

[94] Caretti V., Hiddingh L., Lagerweij T., Schellen P., Koken P.W., Hulleman E., van Vuurden D.G., Vandertop W.P., Kaspers G.J., Noske D.P. (2013). WEE1 kinase inhibition enhances the radiation response of diffuse intrinsic pontine gliomas. Mol. Cancer.

[95] The_UniProt_Consortium UniProtKB—Q13315 (ATM_HUMAN). www.uniprot.org/uniprot/Q13315.

[96] Armata H.L., Golebiowski D., Jung D.Y., Ko H.J., Kim J.K., Sluss H.K. (2010). Requirement of the ATM/p53 tumor suppressor pathway for glucose homeostasis. Mol. Cell Biol..

[97] Lavin M.F. (1998). Radiosensitivity and oxidative signalling in ataxia telangiectasia: An update. Radiother. Oncol..

[98] Li Y., Li L., Wu Z., Wang L., Wu Y., Li D., Ma U., Shao J., Yu H., Wang D. (2017). Silencing of ATM expression by siRNA technique contributes to glioma stem cell radiosensitivity in vitro and in vivo. Oncol. Rep..

[99] Lim Y.C., Quek H., Offenhauser C., Fazry S., Boyd A., Lavin M., Roberts T., Day B. (2018). ATM inhibition prevents interleukin-6 from contributing to the proliferation of glioblastoma cells after ionizing radiation. J. NeuroOncol..

[100] Sinha S., Ghildiyal R., Mehta V.S., Sen E. (2013). ATM-NFkappaB axis-driven TIGAR regulates sensitivity of glioma cells to radiomimetics in the presence of TNFalpha. Cell Death Dis.

[101] Nadkarni A., Shrivastav M., Mladek A.C., Schwingler P.M., Grogan P.T., Chen J., Sarkaria J.N. (2012). ATM inhibitor KU-55933 increases the TMZ responsiveness of only inherently TMZ sensitive GBM cells. J. NeuroOncol..

[102] The_UniProt_Consortium UniProtKB—D6RIG7 (D6RIG7_HUMAN). https://www.uniprot.org/uniprot/D6RIG7.

[103] Brown E.J., Baltimore D. (2000). ATR disruption leads to chromosomal fragmentation and early embryonic lethality. Genes Dev..

[104] Middleton F.K., Patterson M.J., Elstob C.J., Fordham S., Herriott A., Wade M.A., Mccormick A., Edmodson R., May F.E.B., Allan J.M. (2015). Common cancer-associated imbalances in the DNA damage response confer sensitivity to single agent ATR inhibition. Oncotarget.

[105] Yu Z., Xie G., Zhou G., Cheng Y., Zhang G., Yao G., Chen Y., Li Y., Zhao G. (2015). NVP-BEZ235, a novel dual PI3K-mTOR inhibitor displays anti-glioma activity and reduces chemoresistance to temozolomide in human glioma cells. Cancer Lett..

[106] Flynn R.L., Cox K.E., Jeitany M., Wakimoto H., Bryll A.R., Ganem N.J., Bersani F., Pineda J.R., Suva M.L., Benes C.H. (2015). Alternative lengthening of telomeres renders cancer cells hypersensitive to ATR inhibitors. Science.

[107] Heaphy C.M., Subhawong A.P., Hong S.M., Goggins M.G., Montgomery E.A., Gabrielson E., Netto G.J., Epstein J.I., Lotan T.L., Westra W.H. (2011). Prevalence of the alternative lengthening of telomeres telomere maintenance mechanism in human cancer subtypes. Am. J. Pathol..

[108] Jackson C.B., Noorbakhsh S.I., Sundaram R.K., Kalathil A.N., Ganesa S., Jia L., Breslin H., Burgenske D.M., Gilad O., Sarkaria J.N. (2019). Temozolomide Sensitizes MGMT-Deficient Tumor Cells to ATR Inhibitors. Cancer Res..

[109] The_UniProt_Consortium UniProtKB-O14757 (CHK1_HUMAN). www.uniprot.org/uniprot/O14757.

[110] The_UniProt_Consortium UniProtKB-Q683Z8 (Q683Z8_HUMAN). www.uniprot.org/uniprot/Q683Z8.

[111] Wu J., Lai G., Wan F., Xiao Z., Zeng L., Wang X., Ye F., Lei T. (2012). Knockdown of CheckpoInt. Kinase 1 Is Associated with the Increased Radiosensitivity of Glioblastoma Stem-Like Cells. Tohoku J. Exp. Med..

[112] Carrassa L., Broggini M., Erba E., Damia G. (2004). Chk1, but not Chk2, is involved in the cellular response to DNA damaging agents: Differential activity in cells expressing or not p53. Cell Cycle.

[113] Morgan M.A., Parsels L.A., Zhao L., Parsels J.D., Davis M.A., Hassan M.C., Arumugarajah S., Hylander-Gans L., Morosini D., Simeone D.M. (2010). Mechanism of radiosensitization by the Chk1/2 inhibitor AZD7762 involves abrogation of the G2 checkpoInt. and inhibition of homologous recombinational DNA repair. Cancer Res..

[114] Pollack I.F.K.S., Lazo J.S. (1996). Blocking of glioma proliferation in vitro and in vivo and potentiating the effects of BCNU and cisplatin: UCN-01, a selective protein kinase C inhibitor. J. Neurosurg..

[115] Signore M., Pelacchi F., di Martino S., Runci D., Biffoni M., Giannetti S., Morgante L., De Majo M., Petricoin E.F., Stancato L. (2014). Combined PDK1 and CHK1 inhibition is required to kill glioblastoma stem-like cells in vitro and in vivo. Cell Death Dis..

[116] Kruger K., Geist K., Stuhldreier F., Schumacher L., Blumel L., Remke M., Wesselborg S., Stork B., Klocker N., Bormann S. (2018). Multiple DNA damage-dependent and DNA damage-independent stress responses define the outcome of ATR/Chk1 targeting in medulloblastoma cells. Cancer Lett..

[117] Hirose Y.M., Pieper R.O. (2005). Abrogation of the Chk1-mediated G(2) checkpoInt. pathway potentiates temozolomide-induced toxicity in a p53-independent manner in human glioblastoma cells. Cancer Res..

[118] Hirose Y.M., Mirzoeva O.K., Berger M.S., Pieper R.O. (2005). Akt activation suppresses Chk2-mediated, methylating agent-induced G2 arrest and protects from temozolomide-induced mitotic catastrophe and cellular senescence. Cancer Res..

[119] Hauge S., Macurek L., Syljuåsen R.G. (2019). p21 limits S phase DNA damage caused by the Wee1 inhibitor MK1775. Cell Cycle.

[120] Narayan R.S., Gasol A., Slangen P.L.G., Cornelissen F.M.G., Lagerweij T., Veldman H., Dik R., van den Berg J., Slotman B.J., Wurdinger T. (2018). Identification of MEK162 as a Radiosensitizer for the Treatment of Glioblastoma. Mol. Cancer.

[121] Patel P., Sun L., Robbins Y., Clavijo P.E., Friedman J., Silvin C., Van Waes C., Cook J., Mitchell J., Allen C. (2019). Enhancing direct cytotoxicity and response to immune checkpoInt. blockade following ionizing radiation with Wee1 kinase inhibition. Oncoimmunology.

[122] Golden E.B., Pellicciotta I., Demaria S., Barcellos-Hoff M.H., Formenti S.C. (2012). The convergence of radiation and immunogenic cell death signaling pathways. Front. Oncol..

[123] Herskind C., Wenz F., Giordano F.A. (2017). Immunotherapy Combined with Large Fractions of Radiotherapy: Stereotactic Radiosurgery for Brain Metastases-Implications for Intraoperative Radiotherapy after Resection. Front. Oncol..

[124] Van Bijsterveldt L., Durley S.C., Maughan T.S., Humphrey T.C. (2021). The Challenge of Combining Chemo- and Radiotherapy with CheckpoInt. Kinase Inhibitors. Clin. Cancer Res..

[125] Metselaar D.S., du Chatinier A., Stuiver I., Kaspers G.J.L., Hulleman E. (2021). Radiosensitization in Pediatric High-Grade Glioma: Targets, Resistance and Developments. Front. Oncol..

[126] NIH U.S. National Institutes of Health: ClinicalTrials.gov. https://clinicaltrials.gov/.

[127] Sanai N., Li J., Boerner J., Stark K., Wu J., Kim S., Derogatis A., Mehta S., Dhruv H.D., Heilbrun L.K. (2018). Phase 0 Trial of AZD1775 in First-Recurrence Glioblastoma Patients. Clin. Cancer Res..

[128] Van Vulpen M., Kal H.B., Taphoorn M.J., El-Sharouni S.Y. (2002). Changes in blood-brain barrier permeability induced by radiotherapy: Implications for timing of chemotherapy? (Review). Oncol. Rep..

[129] A Study to Assess the Safety and Tolerability of AZD1390 Given with Radiation Therapy in Patients with Brain Cancer. https://clinicaltrials.gov/ct2/show/NCT03423628.

[130] Evaluation of LY2606368 Therapy in Combination with Cyclophosphamide or Gemcitabine for Children and Adolescents With Refractory or Recurrent Group 3/Group 4 or SHH Medulloblastoma Brain Tumors. https://clinicaltrials.gov/ct2/show/NCT04023669.

[131] A Phase 0 Study of AZD1775 in Recurrent GBM Patients. https://clinicaltrials.gov/ct2/show/NCT02207010.

[132] Adavosertib, Radiation Therapy, and Temozolomide in Treating Patients with Newly Diagnosed or Recurrent Glioblastoma. https://clinicaltrials.gov/ct2/show/NCT01849146.

[133] Adavosertib and Local Radiation Therapy in Treating Children with Newly Diagnosed Diffuse Intrinsic Pontine Gliomas. https://clinicaltrials.gov/ct2/show/NCT01922076.

[134] Adavosertib and Irinotecan Hydrochloride in Treating Younger Patients with Relapsed or Refractory Solid Tumors. https://clinicaltrials.gov/ct2/show/NCT02095132.

[135] Targeted Therapy Directed by Genetic Testing in Treating Patients with Advanced Refractory Solid Tumors, Lymphomas, or Multiple Myeloma (The MATCH Screening Trial). https://clinicaltrials.gov/ct2/show/NCT02465060.

